# Drug-resistant oral candidiasis in patients with HIV infection: a systematic review and meta-analysis

**DOI:** 10.1186/s12879-024-09442-6

**Published:** 2024-05-31

**Authors:** Amirreza Keyvanfar, Hanieh Najafiarab, Niki Talebian, Mahdi Falah Tafti, Gelareh Adeli, Zahra Ghasemi, Shabnam Tehrani

**Affiliations:** 1 Infectious Diseases and Tropical Medicine Research Center, Shahid Beheshti University of Medical Sciences, Tehran, Iran; 2 Preventative Gynecology Research Center, Shahid Beheshti University of Medical Sciences, Tehran, Iran; 3 Student Research Committee, School of Medicine, Shahid Beheshti University of Medical Sciences, Tehran, Iran; 4 Faculty of Biological Sciences, North Tehran Branch, Islamic Azad University, Tehran, Iran; 5 School of Medicine, Shahid Beheshti University of Medical Sciences, Tehran, Iran

**Keywords:** HIV, Candida, Drug Resistance, Opportunistic infections, Oral candidiasis

## Abstract

**Background:**

Oral candidiasis (OC) is a prevalent opportunistic infection in patients with human immunodeficiency virus (HIV) infection. The increasing resistance to antifungal agents in HIV-positive individuals suffering from OC raised concerns. Thus, this study aimed to investigate the prevalence of drug-resistant OC in HIV-positive patients.

**Methods:**

Pubmed, Web of Science, Scopus, and Embase databases were systematically searched for eligible articles up to November 30, 2023. Studies reporting resistance to antifungal agents in *Candida species* isolated from HIV-positive patients with OC were included. Baseline characteristics, clinical features, isolated *Candida* species, and antifungal resistance were independently extracted by two reviewers. The pooled prevalence with a 95% confidence interval (CI) was calculated using the random effect model or fixed effect model.

**Results:**

Out of the 1942 records, 25 studies consisting of 2564 *Candida species* entered the meta-analysis. The pooled prevalence of resistance to the antifungal agents was as follows: ketoconazole (25.5%, 95% CI: 15.1–35.8%), fluconazole (24.8%, 95% CI: 17.4–32.1%), 5-Flucytosine (22.9%, 95% CI: -13.7-59.6%), itraconazole (20.0%, 95% CI: 10.0–26.0%), voriconazole (20.0%, 95% CI: 1.9–38.0%), miconazole (15.0%, 95% CI: 5.1–26.0%), clotrimazole (13.4%, 95% CI: 2.3–24.5%), nystatin (4.9%, 95% CI: -0.05-10.3%), amphotericin B (2.9%, 95% CI: 0.5–5.3%), and caspofungin (0.1%, 95% CI: -0.3-0.6%). Furthermore, there were high heterogeneities among almost all included studies regarding the resistance to different antifungal agents (I^2^ > 50.00%, *P* < 0.01), except for caspofungin (I^2^ = 0.00%, *P* = 0.65).

**Conclusions:**

Our research revealed that a significant number of *Candida species* found in HIV-positive patients with OC were resistant to azoles and 5-fluocytosine. However, most of the isolates were susceptible to nystatin, amphotericin B, and caspofungin. This suggests that initial treatments for OC, such as azoles, may not be effective. In such cases, healthcare providers may need to consider prescribing alternative treatments like polyenes and caspofungin.

**Registration:**

The study protocol was registered in the International Prospective Register of Systematic Reviews as PROSPERO (Number: CRD42024497963).

**Supplementary Information:**

The online version contains supplementary material available at 10.1186/s12879-024-09442-6.

## Background

Oral candidiasis (OC) is an infection of the mucous membrane of the mouth caused by *Candida species* [1]. Although *Candida spp.* are commensal fungi, they can invade the oral mucosa in certain conditions [2]. *Candida albicans* is the most common etiologic factor for OC. However, the importance of non-*albicans Candida species* (e.g., *C. glabrata, C. tropicalis, C. krusei, C. dubliniensis, C. parapsilosis, C. guilliermondi, and C. kefyr*) is increasing over time [3, 4]. Poor oral hygiene, smoking, age extremes (infants and elderly), excessive consumption of antifungal agents, malnutrition, and immunodeficiency are predisposing factors for OC [1, 2].

The impaired cellular immunity in people living with the human immunodeficiency virus (HIV) imposes a substantial threat of opportunistic infections [5–7]. OC has emerged as both a first clue for diagnosing acquired immunodeficiency syndrome (AIDS) and an indicator of its severity [8, 9]. OC is the most leading and recurring opportunistic infection in HIV-positive patients with a prevalence ranging from 0.9 to 83.0% [10]. It can manifest in diverse clinical forms in people living with HIV, including pseudomembranous (thrush), erythematous, atrophic, hyperplastic, and angular cheilitis [1, 10, 11].

Unlike other immunocompromised patients, for those with HIV infection, no antifungal prophylaxis for OC is recommended. Whereas the first line treatment for OC in HIV-positive patients is fluconazole [12, 13]. Overall, the resistance pattern to antifungal agents in HIV/AIDS individuals undergoing OC is changing, leading to increasingly serious medical concerns [14–16]. Recurrent infections necessitate the extensive consumption of antifungal agents by those living with HIV/AIDS. Thus, they are at increased risk of drug resistance [17, 18]. On the other hand, the incidence of OC caused by non-*albicans candida spp.* in HIV-positive individuals is increasing [19–21]. Surprisingly, these species have a considerable resistance rate to common antifungal agents. These pathogens can cause invasive infections and result in morbidity and mortality owing to the existence of antifungal resistance, limited drug options, and lack of prophylactic measures [10, 22, 23]. Hence, investigating the antifungal resistance profile of *Candida species* responsible for OC in HIV-infected individuals is critical. It would assist clinicians in selecting the most effective antifungal, preventing impending systemic infections, and directing further research toward innovative alternative treatments [1, 10]. This study aimed to explore the prevalence of drug-resistant oral candidiasis in HIV-positive patients.

## Methods

The study complies with the “Preferred Reporting Items for Systematic Reviews and Meta-Analyses” (PRISMA) statement [24]. It was registered in the International Prospective Register of Systematic Reviews as PROSPERO (Protocol number: CRD42024497963).

### Eligibility criteria

We included English-language observational studies reporting drug resistance to fluconazole, itraconazole, amphotericin B, ketoconazole, 5-flucytosine, nystatin, clotrimazole, caspofungin, miconazole, or voriconazole in *Candida species* isolated from HIV-positive adults suffering from OC. In this study, only publications that reported antifungal resistance in each *Candida* species separately were included. Case reports and case series, review articles, clinical trials, animal studies, commentaries, letters to the editor, guidelines, and conference papers were excluded.

### Search strategy and information sources

PubMed/Medline, Embase, Scopus, and Web of Science were systematically searched for eligible articles published from January 2000 to November 30, 2023. The search strategy was as follows: ((((((((((((candida) OR (candidosis)) OR (candidoses)) OR (candidiasis)) OR (candidiases)) OR (thrush)) OR (moniliasis)) OR (moniliases)) OR (oral candidiasis)) OR (oral candidosis)) AND (((((((oral cavity) OR (oral)) OR (mouth)) OR (palate)) OR (palates)) OR (tongue)) OR (buccal cavity))) AND ((((HIV) OR (human immunodeficiency virus)) OR (AIDS)) OR (acquired immunodeficiency syndrome))) AND (((((drug resistance) OR (antifungal drug resistance)) OR (drug-resistant)) OR (resistance)) OR (resistant)). In addition, all the references in the selected publications were manually searched to identify further studies.

### Study selection

The records found by searching databases were merged, and the duplicates were removed using EndNote X6 software (Thomson Reuters, New York, NY, USA). The records were screened in two rounds. Initially, they were independently screened by two reviewers regarding the title and abstract (MFT and ZG). Then, the full texts of those that passed the initial screening were independently assessed for eligibility by the same reviewers (MFT and ZG). Disagreements were resolved by the principal investigators (ST and AK).

### Data extraction

The following variables were independently extracted from the selected studies by two reviewers (HN and NT): first author name, publication year, country where the study was performed, number of patients, number of isolates, age and sex distribution, current highly active antiretroviral therapy (HAART), CD4 count, history of OC, history of antifungal medication, clinical manifestations of OC, isolated *Candida species*, method of investigating drug resistance, and antifungal resistance pattern. Disagreements were resolved by the principal investigators (ST and AK).

### Statistical analysis

Data were analyzed using STATA software (version 17, IC; Stata Corporation, College Station, TX, USA). The weight of each study in the pooled proportion was the inverse of its variance. The pooled proportion with 95% CI was calculated using the random effect model with restricted maximum likelihood (REML) method or the fixed-effect model. The I^2^ criteria, with a cut-point of 50%, were considered to assess between-study heterogeneity. Publication bias was evaluated by Egger’s test. In this study, the *P*-value < 0.05 was considered statistically significant.

### Quality assessment

The checklist provided by the Joanna Briggs Institute (JBI) was used to perform quality assessment [25].

## Results

### Study selection

**Fig. 1:**
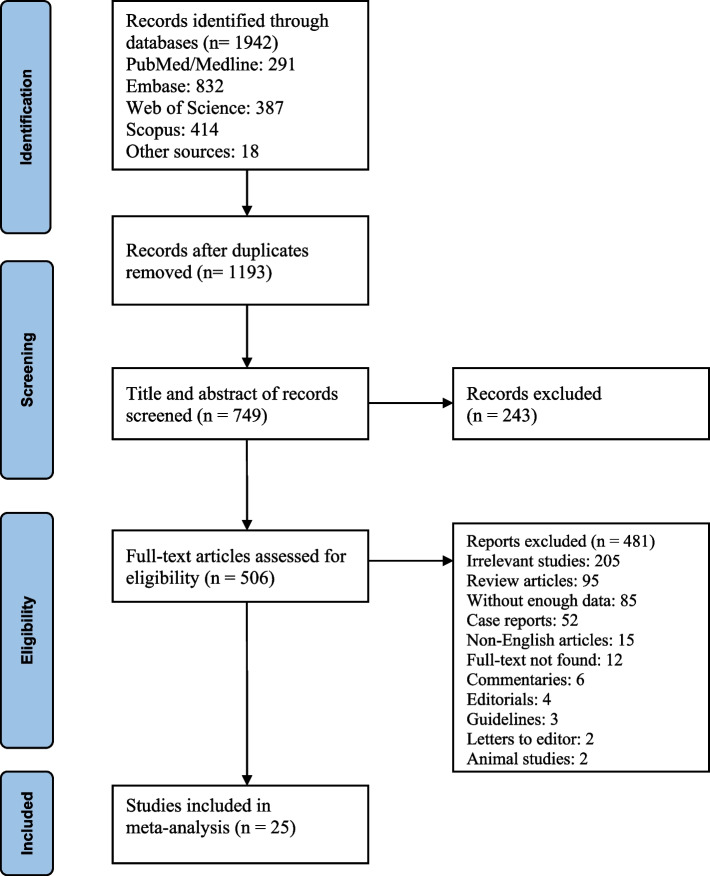
Flow chart of study selection for inclusion in the meta-analysis

Of the 1942 records obtained from an electronic database search, 1193 duplicates were removed. Screening titles and abstracts resulted in the exclusion of 243 records. After assessing the full-text of the remaining records, 25 studies were included for quantitative synthesis and meta-analysis. Figure 1 illustrates the flow chart of study selection for inclusion in the meta-analysis.

### Study characteristics

The detailed characteristics of the included studies are presented in Table 1. The included studies consisted of 2564 *Candida species* isolated from HIV-positive patients with OC. Baseline characteristics and clinical features of the patients are summarized in Table S1. Overall, 48.7% and 36.6% of the patients had a history of OC and antifungal medication, respectively. Pseudomembranous candidiasis (91.6%) was the most common clinical manifestation, while erythematous candidiasis (14.6%), hyperplastic candidiasis (3.9%), angular cheilitis (3.6%), and atrophic candidiasis (1.0%) were less common. Table S2 illustrates the frequency of *Candida species* in different studies. The most frequent species was *C. albicans* (*n* = 1798), followed by *C. glabrata* (*n* = 230), *C. tropicalis* (*n* = 186), *C. krusei* (*n* = 98), *C.dubliniensis* (*n* = 87), *C. parapsilosis* (*n* = 69), *C. gulliniermondii* (*n* = 36), *C. kefyr* (*n* = 27), and *C. famata* (*n* = 17).

**Table 1:** Characteristics of the included studies

First author	Country	Year of publication	Number of patients	Number of isolates	Antifungal susceptibility method	Quality of studies
Magaldi et al. [26]	Venezuela	2000	108	137	Disk diffusion	High
Sant’Ana et al. [27]	Brazil	2002	130	142	Broth microdilution	High
Silva et al. [28]	Brazil	2002	59	59	Broth microdilution	Moderate
Migliorati et al. [29]	Brazil	2004	19	23	Disk diffusion	High
Enwuru et al. [30]	Nigeria	2008	73	74	Broth microdilution	High
Nadagir et al. [31]	India	2008	132	132	Broth microdilution	High
Hamza et al. [32]	Tanzania	2008	292	296	Broth microdilution	High
Jeddy et al. [33]	India	2011	21	21	Disk diffusion	Moderate
Nweze et al. [20]	Nigeria	2011	120	120	Broth microdilution	High
Castro et al. [34]	Columbia	2013	71	93	E-test	High
Katiraee et al. [35]	2013	23	23	Disk diffusion	High	
Gaona-Flores et al. [36]	Mexico	2013	91	91	Broth microdilution	High
Dos Santos Abrantes et al. [37]	South Africa and Cameroon	2014	254	254	Broth microdilution	High
Shyamala et al. [38]	India	2014	118	121	Disk diffusion	Moderate
Katiraee et al. [39]	Iran	2015	NS	83	Disk diffusion	High
Khedri et al. [17]	Iran	2018	89	89	Broth microdilution	High
Murtiastutik et al. [40]	Indonesia	2019	25	25	Disk diffusion	High
Lamichhane et al. [41]	Nepal	2020	25	25	Disk diffusion	High
Ambe et al. [42]	Cameroon	2020	162	171	Disk diffusion	High
Quansah et al. [43]	Ghana	2020	194	194	E-test	High
Tamai et al. [44]	Iran	2021	50	50	Disk diffusion	High
Murtiastutik et al. [45]	Indonesia	2022	23	40	Disk diffusion	High
Erfaninejad et al. [46]	Iran	2023	94	109	Broth microdilution	High
Freitas et al. [47]	Brazil	2023	92	94	Broth microdilution	High
Ekwealor et al. [48]	Nigeria	2023	98	98	Disk diffusion	High

*NS* not specified

### Antifungal resistance patterns of different *Candida species*

Table 2 depicts the antifungal resistance patterns of different *Candida species*. *C. famata* (42.9%), *C. kefyr* (40.0%), and *C.dubliniensis* (18.2%) were mostly resistant to ketoconazole. *C. krusei* (61.1%) and *C. parapsilosis* (30.9%) were mostly resistant to fluconazole. The remaining species were mostly resistant to other azoles as follows: *C. gulliniermondii* to itraconazole (48.5%), *C. tropicalis* to miconazole (45.5%), *C. glabrata* to clotrimazole (43.9%), and *C. albicans* to voriconazole (29.7%). Furthermore, the most sensitive antifungal agent in almost all species was caspofungin.

**Table 2:** Antifungal resistance patterns of Candida species

Antifungal agents	Number of studies	Number of isolates	Sensitive	Susceptible dose-dependent	Resistant
***C. albicans***					
Fluconazole	24	1775	1329(74.9)	97(5.4)	349(19.7)
Itraconazole	14	1187	849(71.5)	113(9.5)	225(19.0)
Amphotericin B	13	931	888(95.4)	12(1.3)	31(3.3)
Ketoconazole	10	691	521(75.4)	59(8.5)	111(16.1)
5-Flucytosine	5	453	383(84.5)	1(0.2)	69(15.2)
Nystatin	7	388	367(94.6)	3(0.8)	18(4.6)
Clotrimazole	5	215	167(77.7)	16(7.4)	32(14.9)
Caspofungin	4	351	339(96.6)	12(3.4)	0(0.0)
Miconazole	3	381	348(91.3)	4(1.0)	29(7.6)
Voriconazole	5	391	266(68.0)	9(2.3)	116(29.7)
***C. glabrata***					
Fluconazole	16	225	110(48.9)	49(21.8)	66(29.3)
Itraconazole	11	165	66(40.0)	48(29.1)	51(30.9)
Amphotericin B	9	154	135(87.7)	4(2.6)	15(9.7)
Ketoconazole	8	102	44(43.1)	20(19.6)	38(37.3)
5-Flucytosine	3	50	49(98.0)	0(0.0)	1(2.0)
Nystatin	4	77	60(77.9)	4(5.2)	13(16.9)
Clotrimazole	2	41	19(46.3)	4(9.8)	18(43.9)
Caspofungin	4	94	78(82.9)	15(16.0)	1(1.1)
Miconazole	3	72	37(51.4)	10(13.9)	25(34.7)
Voriconazole	4	61	55(90.2)	0(0.0)	6(9.8)
**C. dubliniensis**					
Fluconazole	9	87	69(79.3)	7(8.1)	11(12.6)
Itraconazole	6	59	40(67.8)	14(23.7)	5(8.5)
Amphotericin B	6	63	60(95.2)	0(0.0)	3(4.8)
Ketoconazole	1	22	12(54.5)	6(27.3)	4(18.2)
5-Flucytosine	3	25	24(96.0)	0(0.0)	1(4.0)
Nystatin	1	2	2(100.0)	0(0.0)	0(0.0)
Caspofungin	2	36	36(100.0)	0(0.0)	0(0.0)
Miconazole	1	2	2(100.0)	0(0.0)	0(0.0)
Voriconazole	4	38	35(92.1)	2(5.3)	1(2.6)
***C. tropicalis***					
Fluconazole	17	180	119(66.1)	13(7.2)	48(26.7)
Itraconazole	12	125	80(64.0)	18(14.4)	27(21.6)
Amphotericin B	9	100	91(91.0)	3(3.0)	6(6.0)
Ketoconazole	7	79	41(51.9)	8(10.1)	30(38.0)
5-Flucytosine	4	47	43(91.5)	0(0.0)	4(8.5)
Nystatin	3	33	26(78.8)	1(3.0)	6(18.2)
Clotrimazole	1	12	7(58.3)	2(16.7)	3(25.0)
Caspofungin	3	24	22(91.7)	2(8.3)	0(0.0)
Miconazole	3	22	11(50.0)	1(4.5)	10(45.5)
Voriconazole	5	75	56(74.7)	3(4.0)	16(21.3)
**C. krusei**					
Fluconazole	15	90	27(30.0)	8(8.9)	55(61.1)
Itraconazole	11	64	26(40.6)	10(15.6)	28(43.8)
Amphotericin B	7	41	27(65.9)	5(12.2)	9(21.9)
Ketoconazole	7	57	27(47.4)	7(12.2)	23(40.4)
5-Flucytosine	2	11	6(54.5)	2(18.2)	3(27.3)
Nystatin	4	34	23(67.6)	6(17.6)	5 (14.8)
Clotrimazole	2	9	4(44.4)	4(44.4)	1(11.2)
Caspofungin	2	6	6(100.0)	0(0.0)	0(0.0)
Miconazole	3	27	19(70.4)	1(3.7)	7(25.9)
Voriconazole	3	18	14(77.8)	0(0.0)	4(22.2)
***C. parapsilosis***					
Fluconazole	12	68	44(64.7)	3(4.4)	21(30.9)
Itraconazole	7	48	35(72.9)	0(0.0)	13(27.1)
Amphotericin B	6	42	41(97.6)	1(2.4)	0(0.0)
Ketoconazole	5	31	22(71.0)	0(0.0)	9(29.0)
5-Flucytosine	2	29	27(93.1)	0(0.0)	2(6.9)
Nystatin	2	6	6(100.0)	0(0.0)	0(0.0)
Caspofungin	1	2	2(100.0)	0(0.0)	0(0.0)
Miconazole	2	6	5(83.3)	0(0.0)	1(16.7)
Voriconazole	3	36	27(75.0)	1(2.8)	8(22.2)
***C. kefyr***					
Fluconazole	6	27	21(77.8)	0(0.0)	6(22.2)
Itraconazole	4	22	12(54.5)	3(13.7)	7(31.8)
Amphotericin B	3	12	11(91.7)	0(0.0)	1(8.3)
Ketoconazole	1	10	6(60.0)	0(0.0)	4(40.0)
Nystatin	1	1	1(100.0)	0(0.0)	0(0.0)
Caspofungin	2	11	11(100.0)	0(0.0)	0(0.0)
Miconazole	1	1	1(100.0)	0(0.0)	0(0.0)
Voriconazole	3	18	12(66.7)	0(0.0)	6(33.3)
***C. gulliniermondii***					
Fluconazole	4	36	22(61.1)	1(2.8)	13(36.1)
Itraconazole	2	33	17(51.5)	0(0.0)	16(48.5)
Amphotericin B	1	11	11(100.0)	0(0.0)	0(0.0)
Ketoconazole	2	24	14(58.3)	1(4.2)	9(37.5)
5-Flucytosine	1	11	11(100.0)	0(0.0)	0(0.0)
Voriconazole	2	33	23(69.7)	0(0.0)	10(30.3)
***C. famata***					
Fluconazole	4	17	11(64.8)	3(17.6)	3(17.6)
Itraconazole	3	14	5(35.7)	4(28.6)	5(35.7)
Amphotericin B	2	11	9(81.8)	0(0.0)	2(18.2)
Ketoconazole	2	7	1(14.2)	3(42.9)	3(42.9)
5-Flucytosine	1	4	4(100.0)	0(0.0)	0(0.0)
Caspofungin	1	7	7(100.0)	0(0.0)	0(0.0)
Voriconazole	1	3	2(66.7)	0(0.0)	1(33.3)

Values are expressed as frequency (%)

### Meta-analysis of resistance to antifungal agents

**Fig. 2:**
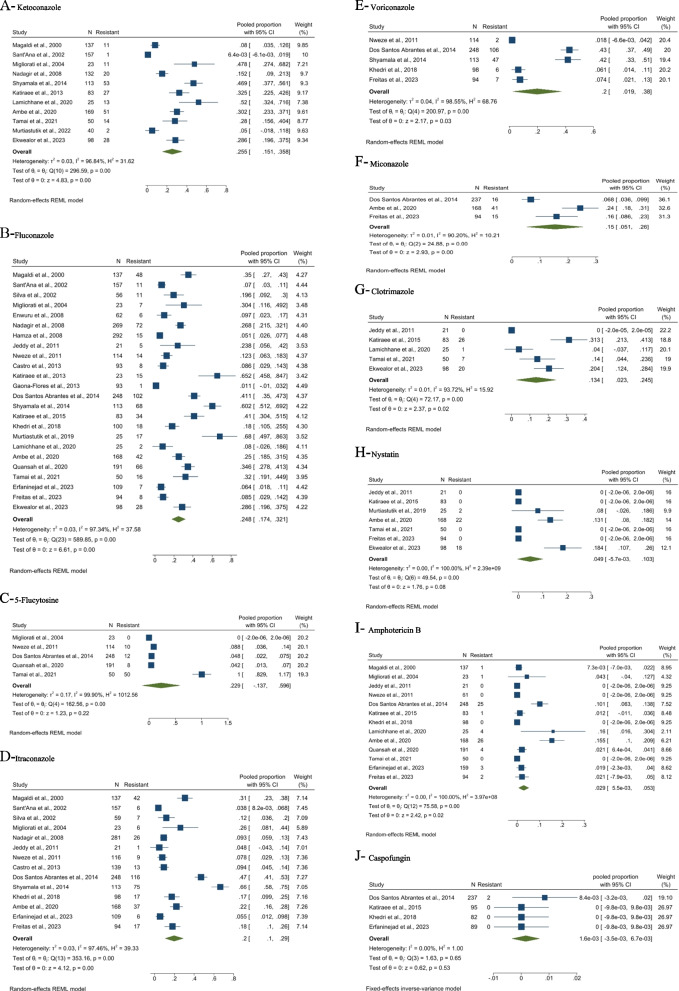
Forest plots of the proportion of anti-fungal resistant oral candidiasis in HIV-positive patients

Figure 2(A-J) demonstrates forest plots of the proportion of anti-fungal resistant OC in HIV-positive patients. The pooled prevalence of resistance to the antifungal agents was as follows: ketoconazole (25.5%, 95% CI: 15.1–35.8%), fluconazole (24.8%, 95% CI: 17.4–32.1%), 5-Flucytosine (22.9%, 95% CI: -13.7-59.6%), itraconazole (20.0%, 95% CI: 10.0–26.0%), voriconazole (20.0%, 95% CI: 1.9–38.0%), miconazole (15.0%, 95% CI: 5.1–26.0%), clotrimazole (13.4%, 95% CI: 2.3–24.5%), nystatin (4.9%, 95% CI: -0.05-10.3%), amphotericin B (2.9%, 95% CI: 0.5–5.3%), and caspofungin (0.1%, 95% CI: -0.3-0.6%).

Furthermore, there were high heterogeneities among almost all included studies regarding resistance to different antifungal agents: ketoconazole (I^2^ = 96.84%, *P* < 0.01), fluconazole (I^2^ = 97.34%, *P* < 0.01), 5-Flucytosine (I^2^ = 99.90%, *P* < 0.01), itraconazole (I^2^ = 97.46%, *P* < 0.01), voriconazole (I^2^ = 98.55, *P* < 0.01), miconazole (I^2^ = 90.20%, *P* < 0.01), clotrimazole (I^2^ = 93.72%, *P* < 0.01), nystatin (I^2^ = 100%, *P* < 0.01), and amphotericin B (I^2^ = 100%, *P* < 0.01). However, studies reporting resistance patterns of caspofungin had no heterogeneity (I^2^ = 0.00%, *P* = 0.65).

### Publication bias

Egger’s test revealed that included studies reporting resistance patterns of ketoconazole (*P* = 0.32), fluconazole (*P* = 0.15), 5-Flucytosine (*P* = 0.11), itraconazole (*P* = 0.21), voriconazole (*P* = 0.14), miconazole (0.20), clotrimazole (0.06), and caspofungin (*P* = 0.20) had no publication bias. Nevertheless, studies reporting resistance patterns of nystatin (*P* < 0.01) and amphotericin B (*P* = 0.01) suffered from publication bias.

## Discussion

In the current systematic review and meta-analysis, we aimed to determine the prevalence of drug-resistant oral candidiasis in HIV-positive patients. Our findings indicated that the pooled prevalence of resistance to azoles and 5-flucytosine was relatively high, ranging between 13.4% and 25.5%. However, over 95% of the isolates were sensitive to nystatin, amphotericin B, and caspofungin. This meta-analysis is the first study to comprehensively report resistance rate to several antifungal agents in HIV-positive patients with OC. Our findings will help clinicians by providing them with knowledge about resistance rates to various antifungal agents, ultimately leading to more effective therapeutic options, reduced treatment failure, and fewer recurrent cases.

There are different classes of antifungal agents available for the treatment of OC, each of which targets a specific cellular component of the fungi. Azoles (e.g., ketoconazole, fluconazole, itraconazole, voriconazole, miconazole, and clotrimazole) inhibit the biosynthesis of ergosterol in the endoplasmic reticulum. Polyenes (e.g., amphotericin B and nystatin) disrupt the membrane structure and function of the fungi by targeting ergosterol in the cell membrane. Pyrimidine analogues (e.g., 5-flucytosine) are converted in the fungi cell to 5-fluorouracil, which inhibits DNA synthesis. And echinocandins (e.g., caspofungin) target fungal cell walls by inhibiting the enzyme β [1, 3]-D-glucan synthase [49, 50].

Our findings revealed that many *Candida* isolates were resistant to azoles, ranging from 13.4% (clotrimazole) to 25.5% (ketoconazole). Nevertheless, many *Candida* isolates were still sensitive to the second-line therapeutic options, such as nystatin (95.1%), amphotericin B (97.1%), and caspofungin (99.9%). Despite the disparities observed in previous studies regarding the prevalence of azole resistance in OC, it is unanimously acknowledged that a significant proportion of *Candida* isolates exhibit resistance to various azoles. They reported the prevalence of azole resistance in *Candida* isolates across the following spectrums: ketoconazole (0.0 [51]-47.8% [39]), fluconazole (4.6 [51]-56.7% [39]), itraconazole (5.4 [46]-66.0% [38]), voriconazole (1.7 [20]-43.0% [37]), clotrimazole (0.0 [33]-38.3% [39]), and miconazole (6.8 [37]-24.0% [42]). The high prevalence of azole resistance may be attributed to cross-resistance to fluconazole, which is routinely administered to HIV-positive patients with clinical manifestations of OC without testing for antifungal sensitivity. Thus, the increased proportion of resistant *Candida spp.* may be caused by prolonged or constant azole administration [47]. The following mechanisms can be employed to make azoles resistant: alteration of the target enzyme (cytochrome P-450 lanosterol 14 α-demethylase) mediated by the ERG11 gene; and failure of azoles to accumulate inside the fungi, followed by enhanced drug efflux mediated by Multidrug resistance (MDR) and Candida drug resistance (CDR) genes [52].

According to the meta-analysis, the pooled prevalence of resistance to 5-fluocytosine was estimated to be 22%. In this regard, 4 out of the 5 studies included in the meta-analysis exhibited a prevalence of 5-fluocytosine resistance close to zero, while only one study from Iran found it at 100%, which skewed the pooled prevalence. Except for the aforementioned article, it can be concluded that most isolates were sensitive to 5-fluocytosine. As reported by Alves et al., flucytosine was more effective against *C. albicans* than *Candida non-albicans* species. Thus, clinicians must consider this matter, when prescribing 5-flucytosine to treat OC [53]. The resistance to this drug is attributed to mutations in the cytosine permease and cytosine deaminase enzymes in *Candida species* [54].

Based on the literature, the minority of *Candida* isolates was resistant to polyenes with the following ranges: amphotericin B (0.0 [44]-16.0% [41]) and nystatin (0.0 [44]-18.4% [48]). According to a World Health Organization (WHO) recommendation in 2014, topical therapy with nystatin suspension would be an alternative to oral fluconazole for treating HIV-positive patients suffering from OC [55, 56]. Although amphotericin B is not the first-line therapeutic option for OC, it may be recommended for patients with fluconazole-refractory OC [17]. The emergence of isolates with polyene resistance raises concerns regarding OC treatment. The resistance to polyenes is achieved by the modification of enzymes involved in ergosterol biosynthesis through ERG2 and ERG3 gene alteration and by the generation of deviate reactive oxygen species (ROS) through overactivated catalase [49].

Unanimously, the prevalence of caspofungin resistance was around zero in the four studies included in the meta-analysis. Caspofungin is an exclusively intravenous antifungal drug. Since most *Candida* isolates are still sensitive to caspofungin, it can be considered as a therapeutic option for refractory or recurrent OC [55].

Furthermore, the prevalence of almost all antifungal agents had high levels of heterogeneity between publications. These heterogeneities may be attributed to temporal variations or differences in the history of antifungal agent administration, drug resistance testing methods, and *Candida species* causing OC [20, 57]. We discussed each of the factors that contribute to the heterogeneities in the following paragraphs.

Osaigbovo et al. reported that 88.9% and 72.8% of resistant isolates were obtained from HIV-positive patients who had utilized fluconazole and had a history of OC, respectively [57]. Recurrent OC and prolonged exposure to antifungal agents resulted in increased resistance of *Candida spp.* to azoles and treatment failures [58]. The overexpression of drug efflux pumps by fungi in response to inappropriate use of an individual azole leads to emerging resistance to multiple agents belonging to the azole family. It could explain the increased resistance to azole antifungal agents [4, 59]. Clinicians can consider fluconazole-resistant *Candida species* as the cause of oral candidiasis in cases of treatment failure or recurrent OC and switch the treatment to alternative therapeutic options [57].

The studies included in our meta-analysis investigated the resistance patterns of *Candida species* using different antifungal susceptibility testing methods (e.g., disk diffusion, broth microdilution, and E-test). These methods are slightly different in detecting susceptibility to antifungal agents, which may lead to heterogeneity [60, 61].

Different *Candida species* have variations in their resistance to a particular antifungal agent [62]. As we found in the systematic review, non-Candida albicans species are more resistant to antifungal agents compared with *C. albicans*, which is explainable based on the genetic characteristics of different species [10, 22, 23]. For example, *C. krusei* possesses an inherent resistance to fluconazole, while *C. glabrata* and C. *famata* species can acquire resistance to fluconazole after the first exposure [19, 32, 50]. Moreover, the co-infection of two or more different *Candida species* may contribute to the development of antifungal resistance in previously sensitive ones, resulting in refractory or recurrent OC [17]. These cases of OC present clinicians with challenges that require further laboratory investigations and the prescribing alternative antifungal agents [63].

Our study had some limitations. Although reviewing multiple databases with appropriate queries, some relevant articles might be unintentionally missed. We included publications written in English, which could lead to a language bias. The limited number of published articles on resistance to a certain antifungal drug might contribute to publication bias.

## Conclusions

Our research revealed that a significant number of *Candida species* found in HIV-positive patients with OC were resistant to azoles and 5-fluocytosine. However, most of the isolates were susceptible to nystatin, amphotericin B, and caspofungin. This suggests that initial treatments for OC, such as azoles, may not be effective. In such cases, healthcare providers may need to consider prescribing alternative treatments like polyenes and caspofungin.

### Supplementary Information

Supplementary Material 1.

## Data Availability

The datasets used and/or analysed during the current study are available from the corresponding author on reasonable request.
