# Supplementary material for: Drug-resistant oral candidiasis in patients with HIV infection: a systematic review and meta-analysis
Source: BMC Infect Dis. 2024 May 31;24:546. doi: 10.1186/s12879-024-09442-6 (PMC11143751; doi:10.1186/s12879-024-09442-6)
Supplement: Supplementary file 1 — Supplementary Material 1. [file 12879_2024_9442_MOESM1_ESM.docx]

| Table S1. Baseline characteristics and clinical features of the patients | | | |
| --- | --- | --- | --- |
| Variables | Number of studies | n/N | % |
| Age  20-39 years  40-59 years  >60 years | 8 | 379/643  228/643  36/643 | 59.0%  35.4%  5.6% |
| Sex  Male  Female | 17 | 902/1710  808/1710 | 52.7%  47.3% |
| Current HAART | 16 | 691/785 | 88.0% |
| CD4 count (cell/mm^3^)  <200  ≥200 | 13 | 362/683  321/683 | 53.0%  47.0% |
| History of oral candidiasis | 4 | 229/470 | 48.7% |
| History of antifungal medication | 10 | 297/811 | 36.6% |
| Clinical manifestations  Pseudomemberanous candidiasis  Erythematous candidiasis  Hyperplastic candidiasis  Angular cheilitis  Atrophic candidiasis | 7 | 663/724  106/724  28/724  26/724  7/724 | 91.6%  14.6%  3.9%  3.6%  1.0% |
| n: number of patients with any variables, N: the total number of studied patients, HAART: highly active antiretroviral therapy | | | |

| Table S2. Frequency of Candida species in different studies | | | | | | | | | |
| --- | --- | --- | --- | --- | --- | --- | --- | --- | --- |
| Studies | ***C. albicans*** | ***C. glabrata*** | ***C. dubliniensis*** | ***C. tropicalis*** | ***C. krusei*** | ***C. parapsylosis*** | ***C. kefyr*** | ***C. guilliemodii*** | ***C. famata*** |
| Magaldi et al. | 108 | 10 | - | 13 | 1 | 5 | - | - | - |
| Sant'Ana et al. | 142 | 9 | - | 4 | 2 | - | - | - | - |
| Silva et al. | 52 | - | - | 4 | 3 | - | - | - | - |
| Migliorati et al. | 17 | 1 | - | 1 | - | - | - | - | 4 |
| Enwuru et al. | 30 | 4 | 1 | 13 | 5 | 3 | 2 | 1 | 3 |
| Nadagir et al. | 90 | - | 22 | 4 | 9 | 5 | - | 2 | - |
| Hamza et al. | 250 | 20 | 1 | 8 | 10 | - | 3 | - | - |
| Jeddy et al. | 19 | - | - | - | 2 | - | - | - | - |
| Nweze et al. | 54 | - | 9 | 22 | - | 18 | - | 11 | - |
| Castro et al. | 70 | 11 | - | 7 | 4 | 1 | - | - | - |
| Katiraee et al. | 23 | - | - | - | - | - | - | - | - |
| Gaona-Flores et al. | 90 | 2 | - | - | - | 1 | - | - | - |
| Dos Santos Abrantes et al. | 194 | 36 | 11 | 4 | 3 | - | - | - | - |
| Shyamala et al. | 15 | 7 | - | 28 | 12 | 16 | 10 | 22 | 3 |
| Katiraee et al. | 60 | 23 | - | - | - | - | - | - | - |
| Khedri et al. | 54 | 9 | 16 | 12 | - | - | 7 | - | - |
| Murtiastutik et al. | 25 | - | - | - | - | - | - | - | - |
| Lamichhane et al. | 25 | - | - | - | - | - | - | - | - |
| Ambe et al. | 103 | 29 | - | 11 | 21 | 4 | - | - | - |
| Quansah et al. | 134 | 13 | 5 | 20 | 8 | 11 | - | - | - |
| Tamai et al. | 50 | - | - | - | - | - | - | - | - |
| Murtiastutik et al. | 23 | 5 | - | 6 | 5 | 1 | - | - | - |
| Erfaninejad et al. | 39 | 26 | 20 | 8 | 3 | 2 | 4 | - | 7 |
| Freitas et al. | 70 | 7 | 2 | 9 | 3 | 2 | 1 | - | - |
| Ekwealor et al. | 61 | 18 | - | 12 | 7 | - | - | - | - |
| Total | 1798 | 230 | 87 | 186 | 98 | 69 | 27 | 36 | 17 |
| Other Candida species were uncommon with the following frequency: *C. lusitaniae* (6), *C. pseudotropicalis* (6), *C. africana* (4), *C. stellatoidea* (3), *C. pelliculosa* (1), and *C.pintolopesii* (1). | | | | | | | | | |
